# Two birds with one stone: How *RhRAP2.4L* controls both cell proliferation and expansion to regulate petal growth in rose

**DOI:** 10.1093/plphys/kiae004

**Published:** 2024-01-10

**Authors:** Janlo M Robil

**Affiliations:** Assistant Features Editor, Plant Physiology, American Society of Plant Biologists; Department of Biology, School of Science and Engineering, Ateneo de Manila University, Quezon City 1108, Philippines

The control of organ size in plants is a complex process involving a coordinated interplay of cell proliferation and expansion. Plant hormones have long been recognized for their pivotal role in this process. For instance, brassinosteroids and gibberellic acid have been demonstrated to modulate growth in eudicot and monocot leaves, respectively (Nelissen et al. 2012; Zhiponova et al. 2013). In contrast, auxin and cytokinins (CK) have been found to regulate growth in roots in an antagonistic manner (Street et al. 2016; Di Mambro et al. 2017). Despite significant progress, molecular mechanisms that link hormone actions with cell proliferation and expansion processes during organ growth remain largely unknown. Filling this knowledge gap is crucial, given that organ growth directly influences biomass, a key factor in food and energy production.

In this issue of *Plant Physiology*, Wang et al. (2023) investigated the role of *RhRAP2.4L*, an AP2/ERF transcription factor, in the regulation of petal growth in rose (*Rosa hybrida*). Its Arabidopsis co-ortholog, *AtRAP2.4b*, was previously identified as a promoter of cell proliferation in response to wounding (Iwase et al. 2011), but the precise mechanisms by which this gene influences organ growth have remained unclear until now. Wang et al. (2023) provided genetic and molecular evidence that *RhRAP2.4L* controls both cell proliferation and expansion to regulate rose petal growth. Moreover, they showed that CK regulates *RhRAP2.4L*, connecting hormone actions with molecular regulators of organ growth.

The authors began by defining the cell proliferation and expansion phases in growing rose petals based on a timeline of changes in cell number and size in the epidermis. They then used RNA-seq to identify genes that were differentially expressed during the early and late stages of petal growth, which correspond to cell proliferation and expansion phases, respectively. Among the genes upregulated in the early stage was *RhRAP2.4L*. Further analyses revealed that *Rh*RAP2.4L acts as a transcriptional repressor and that CK treatment significantly increases its expression. Considering that CK treatment inhibited cell expansion in the petals and that CK levels correlated with *RhRAP2.4L* expression, the authors hypothesized that this gene acts as a CK-responsive repressor, regulating petal growth.

To understand the function of *RhRAP2.4L*, the authors examined petal growth in VIGS-silenced lines. In these plants, *RhRAP2.4L* silencing led to petals with fewer cells, indicating a reduction in cell proliferation. This observation was consistent with lower expression of a cell proliferation marker in the petals. Notably, cell expansion started earlier in *RhRAP2.4L*-silenced plants, indicating that the reduction in cell proliferation resulted from a premature shift of the petals to the cell expansion phase. Therefore, the authors propose that *RhRAP2.4L* plays a role in regulating the timing of the transition from cell proliferation to cell expansion in rose petals.

To dissect how *RhRAP2.4L* modulates the timing of growth transition, Wang et al. (2023) analyzed the transcriptome of early-stage petals from silenced plants, revealing 2 key downstream targets: a cyclin-dependent kinase inhibitor, *RhKRP2*, and a bHLH transcription factor, *RhBPEub*. Subsequent in vitro and in vivo assays revealed that *RhRAP2.4L* binds the promoters and represses the transcription of both genes, indicating that they are direct targets of *RhRAP2.4L*. The co-orthologs of *RhKRP2* in Arabidopsis halt cell proliferation by inhibiting cell cycle progression (De Veylder et al. 2001). In contrast, the authors found that *RhBPEub* levels increased during the cell expansion phase, directly activating genes promoting cell expansion, which is consistent with the known function of its Arabidopsis co-orthologs (Szécsi et al. 2006). Based on these findings, the authors propose that *RhRAP2.4L* regulates the transition from cell proliferation to cell expansion by simultaneously controlling these 2 processes.

This study and a recent study (Jing et al. 2023) have established the role of CK in the regulation of rose petal growth. In the present study, analysis of the *RhRAP2.4L* promoter revealed binding sites for type-B ARRs, which act as positive regulators in the CK signaling pathway. Among the 5 identified type-B ARRs, *RhARR14* was found to be the most likely upstream regulator based on in vivo assays that confirmed its binding and activation of *RhRAP2.4L*. Silencing *RhARR14* resulted in a significant reduction in *RhRAP2.4L* expression. Furthermore, *RhARR14*-silenced plants exhibited an earlier start to cell expansion, as indicated by decreased cell number and an earlier increase in cell size. Therefore, the authors conclude that CK regulates *RhRAP2.4L* via *RhARR14* to control the transition from cell proliferation to cell expansion during petal growth.

In summary, the authors propose that *RhRAP2.4L* acts as a CK-responsive switch, initially promoting cell proliferation by repressing a cell cycle inhibitor, *RhKRP2*. As petal growth progresses and CK levels drop, *RhRAP2.4L* is downregulated, allowing cell expansion to take over by derepressing *RhBPEub*. Their findings support that the dual functions of *RhRAP2.4L* modulate the transition from cell proliferation to expansion, which ultimately determine the size of the petals (Fig. 1).

**Figure 1.**
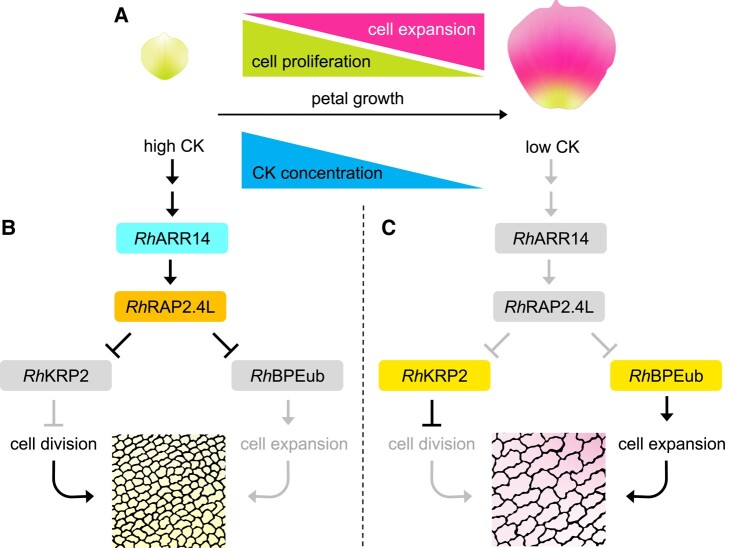
Regulation of rose petal growth by CK and *Rh*RAP2.4L. **A)** Schematic depiction of the progression of rose petal growth from the early stage (left) to the late stage (right). This progression corresponds to the stages of cell proliferation and expansion, respectively, and correlates with a decrease in CK concentration. **B)** During the early stage of petal growth, high levels of CK induce the expression of *Rh*RAP2.4L via *Rh*ARR14. *Rh*RAP2.4L represses both the cyclin-dependent kinase inhibitor, *RhKRP2*, and a bHLH transcription factor, *RhBPEub*, resulting in cell proliferation. **C)** During the late stage of growth, low levels of CK lead to the derepression of *RhKRP2* and *RhBPEub*, which in turn inhibits cell proliferation and promotes cell expansion (Adapted from Fig. 8, Wang et al. 2023).

Wang et al. (2023) introduced a regulatory pathway that orchestrates petal growth in rose, advancing our understanding of mechanisms controlling organ size in plants. Previous studies on meristems and other lateral organs have proposed alternative CK-dependent pathways controlling cell proliferation and expansion. However, Wang et al. (2023) build on this knowledge by demonstrating that a single transcription factor can regulate both processes. This discovery prompts 2 important questions: (1) Is this pathway conserved across different plant organs and plant groups? (2) How does this pathway interact with other hormone-responsive pathways to control organ growth? Future exploration of these questions could lead to an integrative understanding of plant organ growth and the uncovering of tunable mechanisms for the control of organ size in plants.

## Dive Curated Terms

The following phenotypic, genotypic, and functional terms are of significance to the work described in this paper:

plant hormone CHEBI: CHEBI:37848
